# Health system barriers to the implementation of the national action plan to combat antimicrobial resistance in Vietnam: a scoping review

**DOI:** 10.1186/s13756-024-01364-x

**Published:** 2024-01-25

**Authors:** Giang N. Pham, Tho T. H. Dang, Thu-Anh Nguyen, Shukry Zawahir, Hien T. T. Le, Joel Negin, Carmen Huckel Schneider, Greg J. Fox

**Affiliations:** 1 Administration of Science Technology and Training, Ministry of Health, Hanoi, Vietnam; 2 Woolcock Institute of Medical Research, Hanoi, Vietnam; 3 School of Public Health, The University of Sydney, Sydney, Australia; 4 Central Clinical School, The University of Sydney, 90-92 Parramatta Road, Sydney, NSW 2006 Australia

**Keywords:** Health systems barriers, Antimicrobial resistance, National action plan, Vietnam

## Abstract

**Background:**

Vietnam is among 11 countries in the Western Pacific region that has developed a National Action Plan for Antimicrobial Resistance (NAPCA).

**Methods:**

This scoping review characterises health system barriers to the implementation of the Vietnam NAPCA, with reference to the WHO Health Systems Framework.

**Results:**

Over 7 years, between 2013 and 2020, the Ministry of Health (MOH) of Vietnam has been implementing activities to achieve the six NAPCA objectives. They include revision of regulations needed for antimicrobial resistance (AMR) prevention programs; formation and operation of national management bodies; improvement of antimicrobial stewardship (AMS) in hospitals; maintenance of surveillance systems for AMR; provision of trainings on AMR and antibiotics use to doctors and pharmacists; and organization of nation-wide educational campaigns. Limited cooperation between MOH management bodies, shortages of human resource at all health system levels, a low degree of agreement between national and hospital guidelines on antibiotic use, low capability in the domestic supply of standardised drugs, and unequal training opportunities for lower-level health professionals present ongoing challenges. Actions suggested for the next period of the NAPCA include a final review of what has been achieved by the plan so far and evaluating the effectiveness of the different components of the plan. Different options on how to improve coordination across sectors in the development of a new NAPCA should be put forward.

**Conclusions:**

The 6-year implementation of the Vietnam NAPCA has yielded valuable lessons for AMS in Vietnam, guiding the development of future national plans, with a central focus on scaling up AMS in hospitals and promoting community AMS programs to combat AMR.

**Supplementary Information:**

The online version contains supplementary material available at 10.1186/s13756-024-01364-x.

## Introduction

Antimicrobial agents are critical to the treatment of bacterial infections. They also protect patients against infection during a wide range of contemporary medical procedures including surgery, neonatal care, intensive care, transplantation, and cancer treatment [1–3]. However, the overuse and misuse of antimicrobials have attributed to escalating rates of antimicrobial resistance (AMR) [4, 5]. A surge in drug resistance globally over the last 2 decades [4–7] has driven the World Health Organization (WHO) to label AMR as one of the three greatest threats to public health today [8].

The increase in AMR, along with a shortage of new alternatives to antimicrobials, have contributed to the expansion of multidrug resistant (MDR) bacteria which have particularly serious consequences for patients in hospital settings [5, 6]. By 2050, the problem of AMR is estimated to cost £66 trillion and 10 million deaths per annum worldwide [9]. Prime areas for action include AMR surveillance, infection control and antimicrobial stewardship [8].

Vietnam, a middle-income country in Southeast Asia, is a hotspot for the emergence of drug resistant diseases, epitomising the regional challenges facing health systems in combating AMR [10]. Changes to pharmaceutical industry regulation following Vietnam’s “Đổi Mới” economic reforms in the 1980s have contributed to widespread overuse of antibiotics [8, 10]. These reforms led to legalization of private pharmacies, the liberalization of the production and sale of pharmaceuticals and a proliferation of drugstores and pharmaceutical companies. Consequently, a previously universal public health system shifted to a model of largely unregulated public–private antibiotic supply [11]. At the same time, Vietnam continues to experience a high prevalence and incidence of infectious diseases, with substantial associated morbidity and mortality [10, 12]. This combination of factors has contributed to selective pressure upon microorganisms, leading to high rates of drug resistance for a range of pathogens including *M. tuberculosis* [12] , pneumococcus [13], gram negative bacteria [10], and malaria [14]. Surveillance data reveal prevalent antibiotic resistance, where most clinics and hospitals are facing the rapid spread of bacteria. Resistance to a wide range of antimicrobial agents has been reported, including penicillins [15, 16], erythromycin [15, 16], methicillin [15], quinolone [15, 17], lincosamides [15], cefuroxime [18], ceftriaxone [18], amoxicillin/clavulanic [18], gentamicin [18], third and fourth generation cephalosporins [17], and aminoglycosides [17]. The financial and healthcare burdens of AMR are associated with higher treatment costs, prolonged hospitalization, and increased mortality [19].

**Fig. 1:**
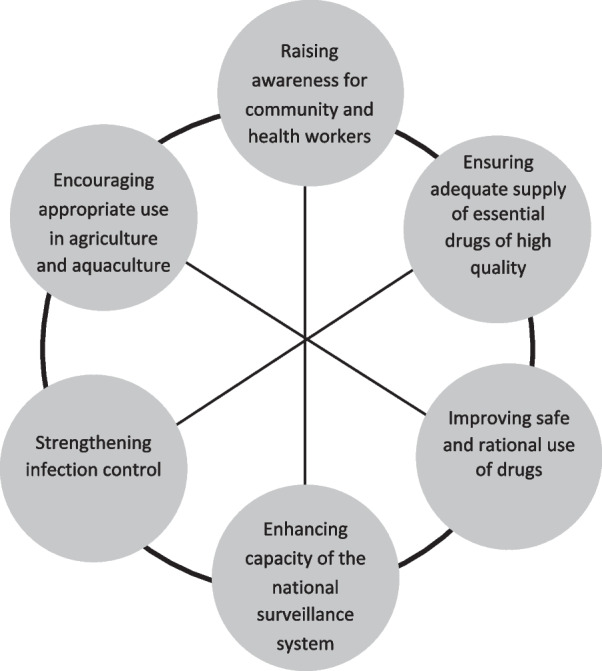
Six blocks of activities in the Vietnam NAPCA [20]

In response to this major public health challenge, the Vietnamese Ministry of Health (MOH) developed a National Action Plan on Combating Antimicrobial Resistance 2013–2020 (NAPCA). The plan included six blocks of activities (Fig. 1) that comprised a detailed plan for strengthening the control of AMR in the country [20]. The strategies to implement the NAPCA include (a) improving policy and management, (b) promoting information, education and communication, (c) reinforcing technical expertise, (d) training the healthcare workforce, (e) financing, and (f) enhancing research and international cooperation [20]. However, little data are publicly available regarding the barriers to the implementation of the current NAPCA that could provide lessons for the development of future interventions into the 2020 NAPCA. In addition, lessons from Vietnam could inform policy and intervention development in other middle-income countries. This scoping review characterises health system barriers to the implementation of the Vietnam NAPCA, with reference to the WHO Health Systems Framework [20, 21] (Fig. 1).

## Methods

The methods applied in this study were qualitative research, using document review and in-depth interviews (IDI). Document reviews were done in advance to provide researchers the initial picture of research topic. This was followed by IDIs with open-ended questions, while continuing to search and review relevant documents. Data collection and analysis was informed by the WHO Health Systems Framework [21]. The current situation and implementation barriers were analysed according to each of the six building blocks of governance, health workforce, medications and technologies, service delivery, health financing, and information.

### Document review

Academic databases (PubMed, the Cochrane central register of controlled trials, and Cochrane database of systematic reviews, Scopus, and Web of Science) were searched from the date of inception, 01 September 2018 until 30 September 2019. To be included in the review, articles needed to meet the following criteria: (1) The content was relevant to antibiotic use and AMR in humans, (2) Health system-related and hospital management factors were mentioned, (3) Involving any level of the Vietnamese healthcare setting, (4) Published within the last 15 years, and (5) English language full text articles were accessible. Attempts were made to contact the lead authors of the documents for which full texts could not be obtained. Medical Subject Headings and relevant search terms of the four key concepts of: (1) health systems, (2) barriers, (3) antimicrobial resistance, and (4) the Vietnam setting were included in the search (see the search strategies in Additional file 1: Table S1). After discarding duplicates, the titles, abstracts, and full-text documents were screened.

The project team examined the physical libraries of the Vietnamese Ministry of Health (MOH) and Vietnamese offices of international institutions including the World Bank, the WHO, the Oxford University Clinical Research Unit (OUCRU) and the Woolcock Institute of Medical Research Vietnam to collect policy documents, regulations, and unpublished project documents and reports. Unpublished works were also sourced via discussions with MOH informants, including reports produced by the MOH departments and national and provincial hospitals which were disseminated at workshops and conferences on AMR.

Peer-reviewed and grey literature, written in English or Vietnamese, were eligible for selection.

### In-depth interviews

Key informants for IDIs were selected through purposive and snowball sampling. The list of key informants for IDIs was prepared based on the management system of MOH on AMR, in which the Medical Services Administration (MSA), with support from the Drug Control Administration of Vietnam and the General Department of Preventive Medicine, was the central coordinator in implementing the NAPCA. In an example of snowball sampling, interviews with two MSA officials responsible for AMR, selected based upon relevant expertise, revealed the critical role of a clinical pharmacist from the Hanoi University of Pharmacy who was later invited to be interviewed. In total there were 38 key informants were invited for IDI and 36 of them were agreed to participate.

Two experienced qualitative researchers (GNP, NTA) conducted the interviews in Vietnamese, using a flexible topic guide covering issues associated with the implementation of and compliance to the NAPCA, the WHO Health Systems Framework, obstacles and challenges associated with implementing the plan, and suggestions for interventions at the state level, in the provinces, and at specific facilities. Notes were taken during in-depth interviews, with the findings were summarized following the IDI, for a debriefing discussion with the others held after completion of every five interviews. Debriefing conferences took place a regular interval between the interviewers to identify emerging themes, refine research questions, and determine whether the findings had reached saturation.

### Data analysis

A thematic coding framework was developed to assist in extracting the key information in line with the six blocks of data in the WHO Health Systems Framework [21] and in each block, we used coding to describe it (Table 1). Selected documents were read thoroughly and coded using this framework. Audio recordings of the IDIs to which the informants gave consent were fully transcribed in Vietnamese. Transcripts were coded in Microsoft Word. The final steps involved discussions of relationships among codes, development of themes, and extended analytical discussions with other co-authors before writing up the report.

**Table 1:** Summarizing the status of implementation of each of the six areas and related barriers to progress

Six blocks of activities	Implementation status	Barriers
Leadership/governance	Released the policy/action plan (NAPCA) Establish working groups Run project 4041	Cooperation among implementation bodies was not good Project objective was not feasible
Health workforce	National manual on antibiotic use, a manual on clinical microbiology testing techniques, and several guidelines on infection control were developed and used in short courses for practicing doctors and pharmacists at national and provincial hospitals	The doctor, pharmacist and other health professionals at the district and lower levels had less chance to get training on AMR topics compared to those working in provincial and national hospitals
Medications and technologies	Government agencies issued regulations indicate that antibiotics only sold under prescription	Antibiotics can still be bought and sold freely in almost all retail pharmacies throughout the country Imported drugs were preferred to locally-manufactured drugs because of the belief that the former are always of better quality
Service delivery	Up to 2019, half of hospitals from national to district levels in Vietnam had formed an AMS team A small number of Vietnamese hospitals have taken proactive steps in introducing AMS to implement the NAPCA in their facilities	The shortages in human resource at both higher- and lower-level hospitals
Health financing	Expenditure on antibiotics comprised one third of total drug costs	Perverse incentives exist that may drive inappropriate antibiotic use
Information	An AMR surveillance system extracting data from project hospitals was established and operated during the period of 2013 to 2016 Communication plan for AMR prevention is issued annually and its objectives are customised for each period	AMR surveillance system experienced delays in data submission from several hospitals to the MOH. Some hospitals did not find the feedback resulted in benefits to them

The study protocol was approved by the Human Research Ethical Committee from the University of Sydney (study number 2018/912) and the Institutional Review Board at the Vietnam National Institute of Hygiene and Epidemiology (granted number IRB-VN01057/IORG 0008555).

## Results

### Search results

Twenty-two peer-reviewed articles relevant to AMR policy in Vietnam were included in the review. Forty-four policy documents and unpublished reports were also included, resulting in a total of 66 documents to be reviewed (Table 2).

**Table 2:** Included literature for review

Type	Quantity	Theme	References
Peer-review articles	22	Health system responses to AMR and antibiotic use	[10, 14–18, 22–37]
Policy documents			
Laws	2	Law of Examination and Treatment and Law on Pharmacy	[38, 39]
Decrees	3	Implementation of the above laws	[40–42]
Circulars	11	Implementation of the above laws and decrees	[43–53]
Decisions	10	Guidance on AMR	[54–63]
National plan	1	The NAPCA	[20]
Guideline	1	Antibiotic management and use	[64]
Reports	6	Pharmaceutical system and health system	[12, 65–68]
Unpublished works			
Reports and presentations	9	Implementation of AMR activities and initiatives	[69–77]
Memorandum	1	Collaborative agreement on AMR	[78]
Total	66		

Five key informants were identified at the national level from MSA and Hanoi University of Pharmacy. Three, which included two MSA officials and one lecturer from the Hanoi University of Pharmacy, agreed to be interviewed in person, and two gave consent to audio recordings. Reasons for refusals included not actually being involved in any AMR-related activities and a busy schedule.

### Leadership/governance

**Fig. 2:**
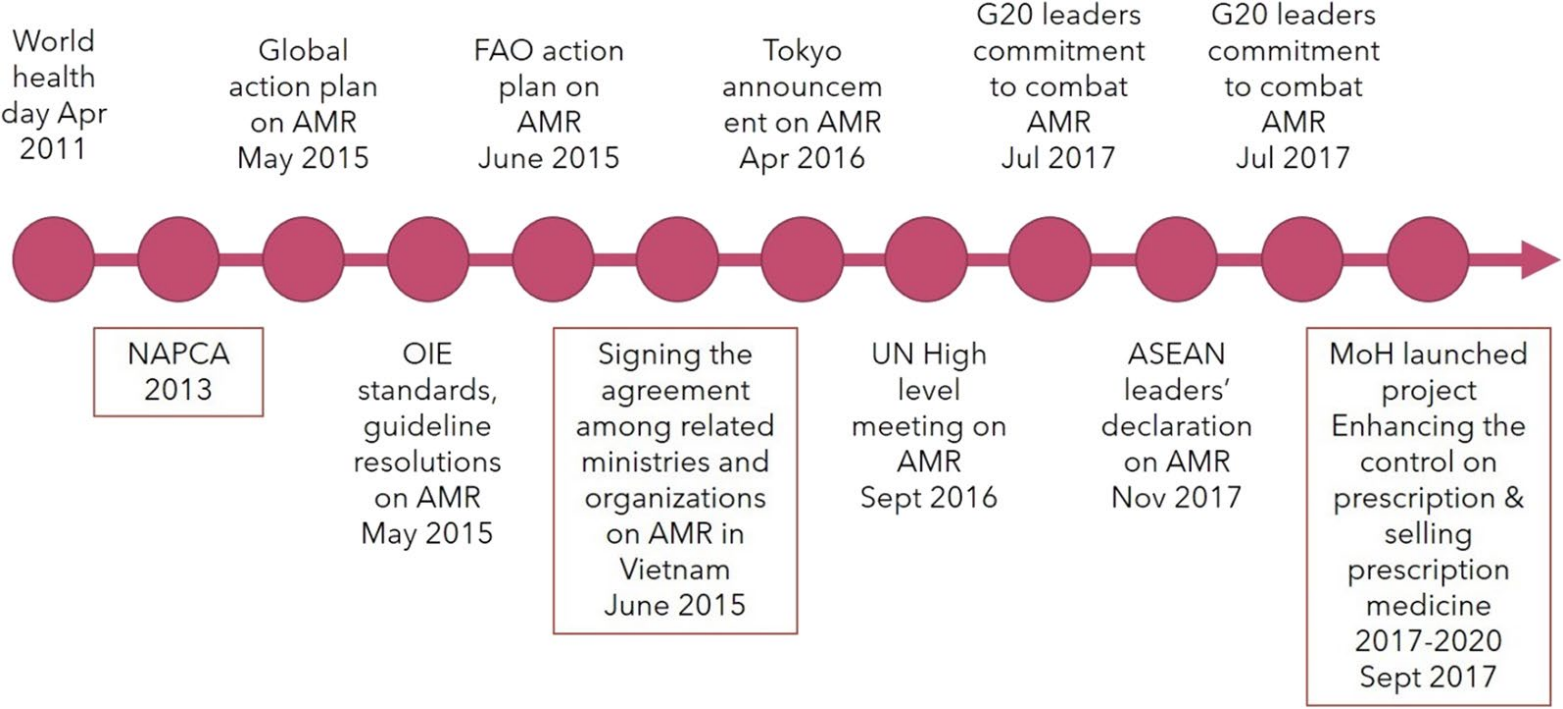
Milestones of international AMR prevention efforts and the respondence of Vietnam (modified from a MSA report) [69]

The MOH of Vietnam is the government agency responsible for coordinating all AMR-related programs in the country. In line with international efforts in AMR, the MOH released the NAPCA in 2013 and later signed an inter-ministerial agreement on AMR in Vietnam (Fig. 2). Since the release of the NAPCA, the MOH nominated several departments to take charge of steering the direction of AMR programs, developing guidance, and implementing activities. In 2014, nine AMR supervision sub-committees were formed to assist the MOH in implementing the NAPCA. A year later, a National AMR Monitoring Unit was established to supervise and monitor the implementation of AMR activities. In 2016, a National Steering Committee on AMR was formed to steer the direction the NAPCA. Each committee employed at least ten officials, but our interviews found that these have not been working full-time on AMR activities. Among them, only three have been permanently involved in the implementation of the NAPCA (*IDI-33*). A MOH mid-term review report on the first phase of NAPCA revealed that the cooperation among these bodies has been sub-optimal and their collaboration has been faced with difficulties and obstacles [69]. The report acknowledged that the AMR supervision sub-committees has not been particularly active in their missions.

In 2017, the MOH launched a project to enhance the state control for prescribing and selling prescription medicine for the period of 2017–2020, viz*.* Project 4041 (see also Fig. 2). The project’s desired outcome is that antibiotics will be 100% dispensed with prescription by 2020—though this aim was not achieved.

Several MOH departments oversee different management responsibilities for the NAPCA. For example, the MSA is responsible for central coordination and is the focal point of running the NAPCA; the General Department of Preventive Medicine oversees the AMR prevention in the community; and the Drug Control Administration of Vietnam supervises the compliance with the Law on Pharmacy. Due to a shortage of skilled staff, the MSA only allocated three officials to work part-time on different aspects of AMR across these departments (*IDI-36*).

### Health workforce

There are no specific policies or regulations on human resources related to AMR, but several regulations issued by the Government and the MOH have directly influenced the human resources allocated to AMR programs. They include Decree No 54/2017/ND-CP that regulates pharmacy professional licensing, including the issuing and withdrawal of practicing licenses for pharmacy practitioners, continuing education on pharmaceuticals, and examinations for obtaining licenses [40]. Circular 02/2018/TT-BYT on Good Pharmacy Practice (GPP) defines the expertise requirements for operating a pharmaceutical retail facility, which requires a facility to have an individual in charge of the pharmacy with professional expertise and an active practice license [43]. The level of degree awarded to that pharmacist determines the scope of business of the retail facility. That is, to run a pharmacy (“nhà thuốc”) the chief pharmacist must hold a Bachelor of Pharmacy (at least 4-year degree). To run a drug counter (“quầy thuốc”) the individual may hold a diploma of pharmacy or equivalent (less than 3 years of education).

**Fig. 3:**
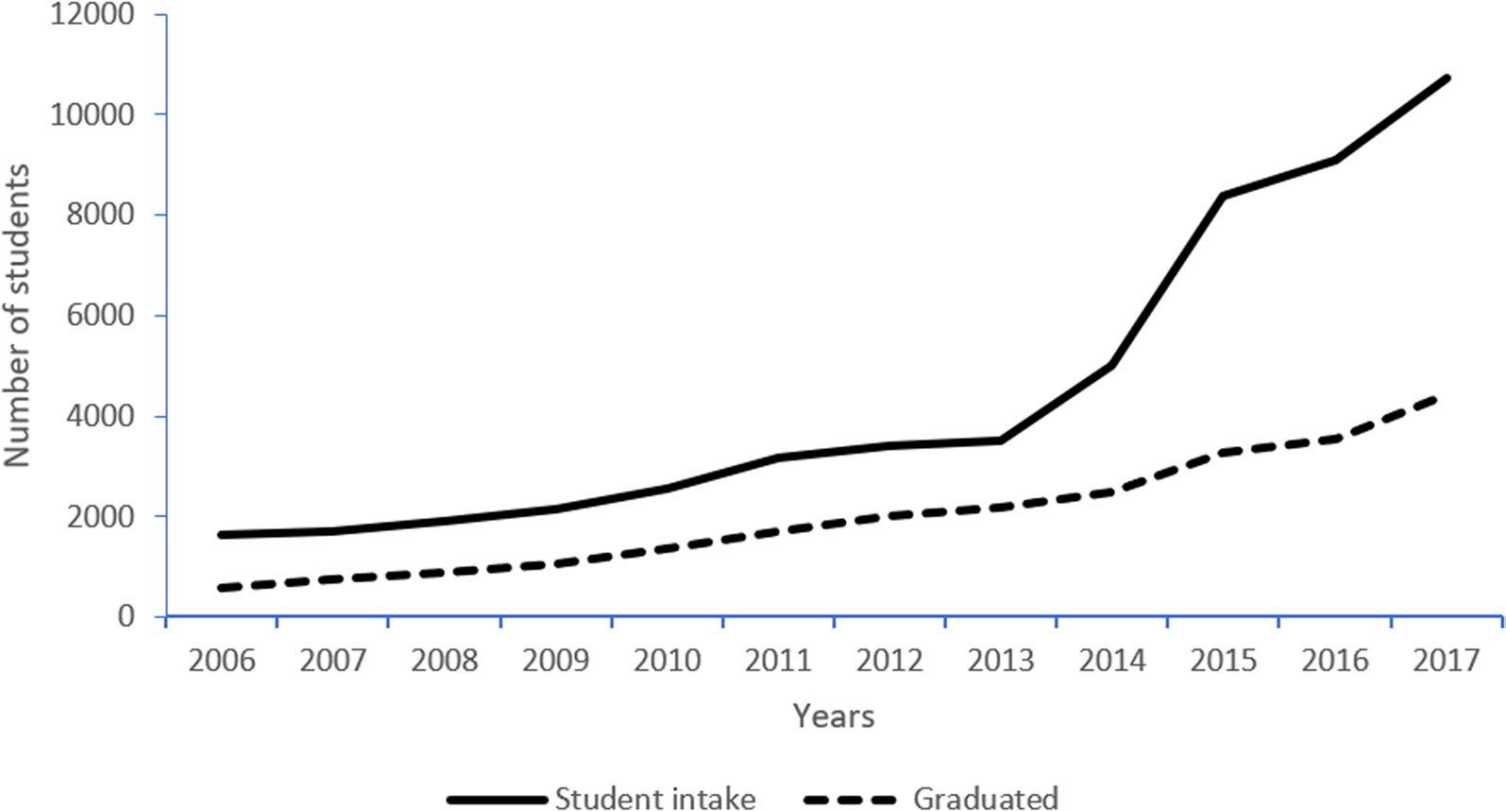
Number of annual intake and graduation from tertiary pharmacy training programs in Vietnam between 2006 and 2017 [62]

Over the past decades, Vietnam has successfully overcome the shortage of health professionals, including undergraduate pharmacists, by encouraging a greater intake of students enrolling in tertiary education in medicine and pharmacy [65]. Apart from establishing new health sciences universities and colleges, private universities such as Duy Tan University (in Danang city), Nguyen Tat Thanh University (in Ho Chi Minh City), and Tay Do University (in Cantho city) have also started pharmacy training programs. As a result, the annual number of pharmacy undergraduates increased eight-fold over a 10 year period (10,716 enrolled students and 4425 graduating pharmacists in 2017 compared to 1630 enrolled students and 590 graduating pharmacists in 2006) (Fig. 3) [70].

Amidst this increase in the training of pharmacists, there is little evidence however that AMR has been included as a core part of the curriculum.

During the first phase of the NAPCA, senior pharmacists and doctors who have expertise on rational drug/antibiotic use in clinical settings from national hospitals, and leading pharmacy and medical universities were invited to join a panel to develop a national manual on antibiotic use, a manual on clinical microbiology testing techniques, and several guidelines on infection control. These manuals and guidelines were used in short courses on AMR for practicing doctors and pharmacists at national and provincial hospitals. The courses were intended to improve the participants’ understanding about antibiotic management, microbiology testing, infectious control, and practical software for antibiotic use management and for WHONET (a free software developed by the WHO to assist management and analysis of microbiology laboratory data) [69, 79]. There are greater training opportunities available for doctors and pharmacists at national and provincial hospitals than for those from district and lower level health facilities. Interviewees noted the need for more training on AMR topics for doctor, pharmacist and other health professionals at the district and lower levels.

### Medications and technologies

To accomplish the NAPCA objectives, the MOH has issued and proposed several policies for regulating the antimicrobial supply chain and pharmacy practices. The first Law on Pharmacy, issued in 2005, authorises antibiotic dispensing only with a prescription [38]. A formal GPP guideline was first released by the MOH in 2007 [54] and revisited in 2011 [44] and in 2018 [43]. Decree 176/2013/ND-CP, issued in 2013, recommended financial penalties for administrative violations against medical laws, with a warning or a fine of between VND200,000 *(US$9)* to VND500,000 *(US$22)* for dispensing of certain prescription drugs (as determined by the MOH) without a prescription [41]. In practice, despite the existence of these laws and regulations, antibiotics can still be bought and sold freely in almost all retail pharmacies throughout the country [22, 23, 80, 81]. The substantial prevalence of unregistered drug outlets in Vietnam poses another significant challenge in the sale of antibiotics without a prescription and proper diagnosis [82, 83]. Scant enforcement has led to low compliance with these policies.

In 2016, the MOH issued an important technical guideline on AMS for hospitals at all levels. Decision 772/QD-BYT required every hospital to establish a formal AMS team to oversee monitoring antibiotic use in the hospital and promoting rational use. Despite this guidance, studies reported an increase in the use of second-line antibiotics, as well as their over-use, at national level hospitals [84, 85]. The degree of correlation between the MOH guidelines and the local hospital empirical guidelines about antibiotic use is low. A patient population in which local guidelines for antibiotic use is particularly discrepant is children under 2 months having a viral illness, in which antibiotic use is common [24]. An increase in the use of colistin, the second-line for MDR gram-negative infections, saw 62.1% of patients prescribed with colistin given without microbiology testing to establish resistance to other antibiotics. These patients were not offered the recommended loading dose of colistin and were frequently given a low maintenance dose. Kidney function was also not monitored, despite its recommendation for patients taking colistin [25]. A study among patients using mechanical ventilation noted that antibiotics were often prescribed for post-intubation, even those without infections [26]. Another study at a national hospital showed a high level of carbapenem use—continuously increasing from 1385 Defined Daily Dose (DDD) per 100 patient-days in 2012 to 2475 DDD per 100 patient-days in 2016 [27].

At the provincial level, reports showed non-compliance with the required dosage and microbiology testing results in the use of many antibiotics. On average, three quarters of antibiotics prescribed (73% of the original regimens and 71% of alternative regimens) complied with the MOH guideline. However, 79% of antibiotics were used outside the range of recommended dosage range, across all classes of antibiotics [28].

A review of international and local evidence on antibiotic use and AMR during the period of 1990 to 2012 showed that half of all drugs consumed in Vietnam were locally produced. Antibiotics accounted for more than half of those drugs—including 70% of drugs used in agriculture [10]. However, Vietnam is still not capable of producing raw medicinal materials for many drugs and has relied heavily on China (51%) and India (18%) for their manufacture. One study showed [71] that in the Vietnamese pharmaceutical market, popular low-cost pharmaceuticals were mainly offered by local manufacturers, while the market of high-value specialised drugs were dominated by foreign companies. Using cheap and substandard antibiotics may lead to reduced treatment efficacy, longer hospital stays, and increased risks of AMR [69]. A study on pharmacy customers’ drug use behaviours in Ho Chi Minh City (HCMC) conveyed that imported drugs were preferred to locally-manufactured drugs because of the belief that the former are always of better quality [29]. Annually, the state drug quality control system run by the MOH Drug Control Administration has checked 35,000 batches of drugs, on average, for quality. This helped to decrease the rate of unqualified drugs [30]. Violations of the standards have been penalised in accordance with the Pharmacy Law, together with additional penalties such as the cancellation of operating licenses, the suspension of importation permits, and having the drug license withdrawn from violating companies [30]. Despite this, the quality of antibiotics available in the market has still not been well-controlled.

### Service delivery

Since 2013, the MOH has encouraged each hospital from national to district level to establish its own Drug and Treatment Council. These bodies have important regulatory roles, such as enforcing management and use of drugs within the hospital, composing the list of available drugs, identifying and analysing problems related to drug use, developing and implementing clinical treatment guidelines, monitoring and reporting adverse drug reactions and treatment errors inside the hospital and to National Drug Information and ADR Center, and disseminating and managing drug information [45]. In 2016, the MOH issued guidance on antibiotic use in hospitals. This regulation required that hospitals at every level establish an AMS team. Team membership included members of the Drug and Treatment Council. These teams took charge of monitoring antibiotic use within the hospitals, implementing interventions to encourage appropriate use of antibiotics, and reporting the quantity of antibiotics used and antimicrobial resistance levels at the institutional level [64]. Three years after introducing this guidance, only half of hospitals from national to district levels in Vietnam had formed an AMS team according to participant interviews. According to interviewees, common reasons for not having this team are the shortages in human resource at both higher- and lower-level hospitals.

Appropriate antibiotic use has been a longstanding challenge in Vietnamese health facilities. The literature has presented two approaches to decision-making about antibiotic use that are commonly followed by doctors in Vietnamese hospitals: (a) experience-based (i.e., based upon the historical practice of doctors and their colleagues), and (b) guideline-based approaches (following local or national guidelines). Experience-based decision-making has typically been employed when doctors had not established the disease etiology and were waiting for results from microbiological tests. A study among pneumonia patients admitted to the ICU department of a provincial general hospital reported that more than half of patients had to switch from the initial empirical antibiotic choice to other antibiotics after obtaining a positive microbiological test result. Selection of appropriate treatment remained a challenge for clinicians even when a diagnosis was reached, since the patients’ microbiological testing results and clinical symptoms were not always concordant [31]. Another study about initial antimicrobial selection conducted at a national level hospital found that just 30.1% patients were prescribed antibiotics in compliance with local guidance [24]. Lower adherence with guidelines was also seen in pediatric patients under 2 months with mild pneumonia.

A small number of Vietnamese hospitals have taken proactive steps in introducing AMS to implement the NAPCA in their facilities. In HCMC, the largest city in Vietnam, the Department of Health (DOH) developed a city Action Plan on AMR prevention based on the NAPCA [72]. Up to 2017, this city had developed a database of 3399 approved treatment regimens that applied to all specialties and facilities [72]. An antibiotic guideline was developed based upon data on microbiological isolates collected within the city hospitals. The approach offers a positive example for other settings.

One example of successful implementation of AMS policies was Nhan Dan Gia Dinh hospital, in Ho Chi Minh City. Real-time supervision of appropriate prescriptions has also been introduced to Nhi Dong I hospital, and computer monitoring has been implemented to require an appropriate “antibiotic indication” before antibiotics can be dispensed at Thu Duc hospital. The HCMC DOH reported it was planning to conduct ongoing inspection and monitoring of pharmacies in dispensing practices for prescribed medicines. Hence it has piloted a “clinical microbiology” model of care—which involves developing local expertise in antibiotic guidance. It is also drafting campaigns for strengthening the awareness of AMS among health professionals and the community.

Cho Ray Hospital, a national-level hospital located in HCMC, has also established its own AMS program, which includes antibiotic use monitoring and especially for surgical patients in which prophylactic antibiotics are frequently inappropriately given [32, 73]. The hospital’s guidance on antibiotic use has been updated frequently, with internal training for healthcare staff based upon these guidelines. Compliance with these guidelines increased by approximately 15% in 2017 compared to that in 2015 [32].

In Hanoi, the National Hospital of Tropical Diseases established an Antimicrobial Resistance Surveillance team to monitor doctors’ prescribing of antibiotics using the hospital management database and patients’ records [74]. The hospital also prepared a guideline on antibiotic use, updated the its diagnostic and treatment guidelines, and applied the classification of antibiotic use in the hospital database. It has also carried out a national research study on antibiotic resistance and cooperated with mass media to produce a television show entitled “The correct use of antibiotics”. A private hospital, Saint Paul Hospital, has also been monitoring pre-operative antibiotic prophylaxis, organizing training to increase health workers’ awareness on the use of narrow-spectrum antibiotics, limiting inappropriate combinations of antibiotics during initial treatment, and switching patients from intravenous to oral antibiotics once clinical stability has been established [75]. Nonetheless, inappropriate antibiotic use persists in hospitals, notwithstanding various efforts to alter the practice through AMS strategies. Interviewees identified a shortage of human resources and funding as pervasive factors contributing to this practice, both at higher- and lower-level hospitals.

### Health financing

The average annual medical expenditure per capita in Vietnam has increased steadily, from US$9.9 in 2005 to US$22.5 in 2010, to US$34.5 in 2014 [66] and US$180.7 in 2019 [86]. Government reimbursements for medicines have always occupied the largest proportions of this cost, ranging from 48.7 to 61.0% among examination and treatment services covered by the national health insurance scheme. Drug costs remain a large part of the total expenditure for medical services [76]. In 2010, hospitals spent 58% of their routine expenditure on medicines [87]. Expenditure on antibiotics comprised one third of total drug costs [77].

Perverse incentives exist that may drive inappropriate antibiotic use. Prescribing antibiotics can bring incentives to individual prescribers, in the forms of financial commissions, funding to attend conferences, gifts, and positive feedback from patients. Interviewees indicated that clinics and hospitals may also receive funding for infrastructure upgrading, and hospitals may receive commissions from their drug sales [10]. Private pharmacies also rely upon antibiotic sales for their financial viability creating incentives for overuse that are not conducive to AMS [88].

### Information

Several channels for health information associated with AMR prevention have been set. In late 2012, 16 large national and provincial hospitals across Vietnam partnered with the Oxford University Clinical Research Unit (OUCRU) and Linköping University in the Vietnam Resistance (VINARES) project, aimed at developing the country’s capacity to deal with AMR including establishment of a national reference lab and surveillance network [33]. A Clinically Oriented antimicrobial Resistance surveillance Network (ACORN) was also piloted in Vietnam and is now being scaled up to other hospitals to provide hospitals with more useful data for clinical decision-making on AMR [89]. Hospitals were equipped with resources from international donors to establish their own AMS programmes. This included monthly data reporting about antibiotic consumption, infection control surveillance, and testing of local susceptibility of bacterial pathogen. These reports were reviewed by programme experts, with feedback given to the hospitals. Hospitals were also offered data about their performance relative to other hospitals, to motivate quality improvement [33].

As one part of the NAPCA, an AMR surveillance system, inherited from the VINARES project (2012–2013), extracting data from project hospitals was established and operated during the period of 2013 to 2016 [34]. Hospital laboratories were also asked to collect data as indicated in the surveillance protocol and submitted monthly to MSA for analysis. To support this system, the MOH issued Circular 33/2016/TT-BYT, which regulated the activities of hospitals’ microbiology laboratories and mandated the use of WHONET to report AMR to the central government’s MSA [46]. International organisations have also played a role in local AMS. The US CDC and PATH Vietnam have supported the MOH in improving the protocol for above mentioned AMR surveillance, as an interviewee reported. Following the protocol, the MSA and its partners have helped to standardise the information collected about AMR from the 16 hospitals’ laboratories. This has helped to determine where local technical support was needed. Technical consultation has also been provided to hospitals to identify issues that could be improved by undertaking small-scale interventions. For example, a local AMS program was introduced in Hue Central Hospital according to one interview participant. Furthermore, a strategy to prevent carbapenem-resistant Enterobacteriaceae (CRE) has been initiated at the HCMC Medical and Pharmacy University Hospital. However, an interviewee reported that this AMR surveillance system experienced delays in data submission from several hospitals to the MOH. Some hospitals did not find the feedback resulted in benefits to them.

Nationally, health promotion has been an important strategy for encouraging appropriate consumer behaviours [69]. A communication plan for AMR prevention is issued annually and its objectives are customised for each period. The WHO also initiated an annual World Antibiotic Awareness Week campaign that aims to increase awareness of global AMR and to encourage best practices among the general public, health workers and policy makers to avoid the further emergence and spread of AMR [12]. This has included talk shows on national television addressing antibiotic use and AMR. The website includes a link for healthcare workers and members of the community to sign a commitment on safe and appropriate use of antibiotics. The national Ministry has also produced leaflets, posters, and banners with slogans such as “No action today, no cure tomorrow” (Không hành động hôm nay, ngày mai không thuốc chữa), “Only dispensing antibiotics with doctors’ prescription” (Bán thuốc kháng sinh phải có đơn của y, bác sỹ). Local AMR prevention campaigns have also been held annually in the provinces of Hanoi, HCMC, and Vinh Phuc province. These have included local and international partners including the WHO, the UN Food and Agriculture Organization, OUCRU, CDC, PATH, British Embassy, French Embassy, Belgium Embassy and other relevant ministries.

## Discussion

Vietnam is one among 11 countries in the Western Pacific region that has developed a National Action Plan for AMR [90], however, major challenges, such as the limited cooperation between national management bodies, a shortage of human resources at all health system levels, and a low degree of agreement between the national and the hospital treatment guidelines remain.

### National consensus, but barriers to local implementation remain

AMS is an important component of the Vietnam NAPCA, which became compulsory in hospitals in Vietnam since the issue of Decision 772/QD-BYT in 2013 (Decision 772) [64]. Though the mid-term review of the NAPCA in 2017 reported some positive effects of the AMS programs—with a reduction in the prevalence of AMR at some national hospitals and in other large hospitals in Hanoi and HCMC [90]. This positive finding is similar to that what is achieved in other countries when AMS programmes were introduced [13, 91]. However, inappropriate antibiotic use persists in hospitals, despite AMS guidelines, due to a range of factors: limited awareness and education among healthcare providers, patient and family pressure for antibiotics, easy over-the-counter access to antibiotics, resource constraints hindering diagnostics, regulatory and enforcement gaps, cultural influences, financial incentives, lack of local resistance data, inconsistent guideline enforcement, and rural–urban healthcare disparities. These factors collectively contribute to the continued inappropriate use of antibiotics in the Vietnamese healthcare system.

However, 3 years after the introduction of the Decision 772, only 50% of hospitals had established the AMS teams, according to an interviewed MSA official. This number was lower than it was in the US, where 71% of hospital had AMS programs in 2014 [92] and in China where 95% hospitals had AMS programs [93]. Despite the fact that the AMS guidelines introduced in Decision 772 applied to all hospitals, localised guidelines with prioritised activities for each specific level of the health system, especially in lower level facilities, are not available. In general, most NAPCA activities have occurred in large cities and in large hospitals with relatively fewer actions taken place in district or community facilities. Treatment guidelines for infections for which antibiotics are frequently used have been developed for all health facilities in Vietnam. Hospitals have been advised to customise the guidelines according to their clinical context, although limited local data is often a barrier to this occurring [94]. As a result, the differences between AMS Ministry of Health guidelines and local empirical guidelines can be attributed to various factors, such as regional variations in disease prevalence and resistance patterns, disparities in resource availability, adaptation to local antibiotic resistance patterns, the influence of healthcare professionals' clinical experience, logistical considerations, and the impact of local regulations and legal requirements. To address these disparities, healthcare institutions should regularly update their guidelines, incorporating the latest evidence and local epidemiological data, with the involvement of a multidisciplinary team to ensure evidence-based guidelines that align with both local context and broader national or international recommendations whenever possible.

Hence, national consensus guidelines for provincial and district hospitals should be a priority given the high proportion of community dwellers being treated at these primary care levels. It would be a major barrier for the validity, reliability, and consistency of the national AMR data if hospitals were to collect data without a uniform system.

### Limited surveillance hinders progress

AMR surveillance has been shown to play an important role in many developed countries [95]. Vietnam adapted this approach to its own AMR surveillance system. The established core platform for data reporting, adapted from the VINARES project, has helped policy-makers to bridge the policy-action gaps that is present in many low- and middle-income countries (LMICs) [96]. Lower level surveillance is also an important area for the NAPCA to address because having surveillance only at the high level may overestimate AMR, since it is taken within the tertiary settings, including ICUs where second line antibiotics are used very frequently. If guidelines are based upon these results, a biasing of reporting towards tertiary facilities may also lead to inappropriate use of second-line antibiotics in lower-level facilities. If a comprehensive surveillance system from both lower and higher level of the healthcare system can be developed, the Vietnamese government will be able to improve the AMR policies. Vietnam is also not yet a contributor to the WHO Global Antimicrobial Resistance Surveillance System (GLASS), despite being a member since 2015 [97].

### Human resources required to drive improvement

Human resource constraints remain an important barrier to implementation of AMS [98]. The shortage is experienced in the national management agency leading to inconsistent coordination and supervision in facilitating the NAPCA activities, and in the hospitals leading to limited implementation of AMS programs. The provision of training on AMR to doctors, pharmacists and other health care professionals who play critical roles in antimicrobial prescription and dispensation has been identified as an important strategy in combatting AMR [91, 99, 100]. Many hospitals in Vietnam have provided training on AMR and antibiotic management in combination. Policies that enable ongoing supervision are critical to improvements in appropriate antibiotic use [32].

For any AMS team, a (clinical) pharmacist plays a vital role [99]. The steady increase in the number of pharmacy graduates opens opportunities to reinforcing AMS in both hospital and community settings [101–103]. However, specific training regarding appropriate dispensing and AMR is still lacking for many graduates. It however can potentially create competitive pressure for sales, which may drive excess antibiotic use in the private sector. Because the criteria required to open a pharmaceutical salespoint are relatively easy to achieve, and there is a plethora of places to purchase antibiotics retail, the drug market is highly competitive. In turn, these pharmacies are driven to make a greater number of sales to remain economically viable.

### Importance of health literacy and community engagement

While numerous efforts have been undertaken to enhance community-level awareness and prevent AMR, the persistently limited knowledge within the community regarding the proper use of antibiotics remains a significant component of the AMR challenge [29, 94, 104–106].

The ineffectiveness of AMR health promotion campaigns in Vietnam can be attributed to limited information accessibility, cultural and linguistic barriers, varying health literacy levels, inadequate resources, persistent antibiotic misuse, a lack of sustainable strategies, insufficient monitoring and evaluation, and the complexity of the AMR message, requiring tailored and simplified communication approaches.

In addition to lack of health literacy regarding AMR. This discrepancy persists knowledge and behaviour of patients mainly due to patients enjoy affordable and convenient access to pharmaceutical care via self-medication practice and pharmacists benefit from selling antibiotics and other health supplements [10]. Relatively small fines for violations of selling antibiotics without a prescription and financial incentives for antibiotic use from pharmaceutical companies encourages sellers to keep dispensing antibiotics inappropriately. This ecosystem of perceived mutual benefit reinforces current inappropriate practices. The importance of having AMR programmes in the community or primary care setting that are aligned with hospital programmes should be acknowledged [107]. In addition to strict policy changes in regards to access to antibiotic, improved continuous education for the public and community pharmacists and pharmacy attendants is an essential prerequisite to enhance the sustainability of AMR programmes [108].

### Recommendations for future action

Vietnam is continuing with the current NAPCA for the period 2022–2023, with a new national plan currently under development. We suggest actions for the next period, including a deep review of what has been achieved by the NACAP to date by independent bodies. This will include monitoring its outcome indicators and evaluating the past effectiveness of the plan. Vietnam can also draw upon the experience internationally. Collaboration between Vietnam and other countries is likely to contribute to future success. Key stakeholders will include international governments, academia, faith-based and non-governmental organizations [109], as well as clinicians and patients. A range of different options should be developed to help guide the new national action plan. As these plans are developed, the MOH should ensure that its programs are evaluated using rigorous study designs and that study results are made widely available. Strategies that address the health system drivers of inappropriate behaviours will be essential, since inappropriate use largely stems from dysfunction within parts of the health system [33].

### Study strengths and limitations

To our knowledge this is the first study that synthesized evidence about the implementation of the NAPCA in Vietnam. The study evaluates the successes of the NAPCA in understanding the local situation, in developing and implementing the plan. It draws upon a wide range of sources, including local policies, respondent interviews and national strategy documents. Nevertheless, a potential limitation of our approach is the use of non-peer-reviewed documents, such as workshop presentations, in which the evidence may not be robust. Another weakness is a possible omission of local initiatives that have not yet been reported or published.

## Conclusions

The 6-year implementation of the Vietnam NAPCA has taught several valuable lessons about AMS in Vietnam. These lessons will help to guide Vietnam as it develops its next national plan. Scale-up of AMS must play a central role in antibiotic management in hospitals at all levels, as well as promotion of community AMS programs to reduce AMR in Vietnam and in the region.

### Supplementary Information

**Additional file 1**. Search strategies.

## Data Availability

Data sharing is not applicable to this article as no datasets were generated or analysed during the current study.

## Abbreviations

AMR: Antimicrobial resistance
AMS: Antimicrobial stewardship
CRE: Carbapenem-resistant Enterobacteriaceae
DDD: Defined daily dose
DFAT: Department of Foreign Affairs and Trade
DOH: Department of Health
GLASS: Global Antimicrobial Resistance Surveillance System
GPP: Good pharmacy practice
HCMC: Ho Chi Minh City
IDIs: In-depth interviews
LMICs: Low- and middle-income countries
MDR: Multidrug-resistant
MOH: Ministry of Health
MSA: Medical Services Administration
NAPCA: National Action Plan for Antimicrobial Resistance
VINARES: Vietnam Resistance
WHO: World Health Organization

## References

[1] Australian Commission on Safety and Quality in Health Care. Antimicrobial Stewardship in Australian Health Care 2018. Australian Commission on Safety and Quality in Health Care, Sydney, Australia; 2018.

[2] Coulter S, Merollini K, Roberts JA, Graves N, Halton K (2015). The need for cost-effectiveness analyses of antimicrobial stewardship programmes: a structured review. Int J Antimicrob Agents.

[3] Mamun A, Zhao B, McCabe M (2019). Cost-benefit analysis of a population-based education program on the wise use of antibiotics. Can J Public Health Revue canadienne de sante publique.

[4] Boucher HW, Talbot GH, Bradley JS (2009). Bad bugs, no drugs: no ESKAPE! An update from the Infectious Diseases Society of America. Clin Infect Dis Off Publ Infect Dis Soc Am.

[5] Ruiz-Ramos J, Frasquet J, Romá E (2017). Cost-effectiveness analysis of implementing an antimicrobial stewardship program in critical care units. J Med Econ.

[6] Nowak AM, Nelson ER, Breidenbach LJ, Thompson AP, Carson JP (2012). Clinical and economic outcomes of a prospective antimicrobial stewardship program. Am J Health Syst Pharm.

[7] Spellberg B, Blaser M, Guidos RJ (2011). Combating antimicrobial resistance: policy recommendations to save lives. Clin Infect Dis Off Publ Infect Dis Soc Am.

[8] World Health Organization (2015). Global action plan on antimicrobial resistance.

[9] O’Neill J. Review on antimicrobial resistance: tackling a crisis for the health and wealth of nations. Wellcome Trust and the UK Government, UK; 2014.

[10] Nguyen KV, Thi Do NT, Chandna A (2013). Antibiotic use and resistance in emerging economies: a situation analysis for Viet Nam. BMC Public Health.

[11] Angelino A, Khanh DT, An Ha N, Pham T (2017). Pharmaceutical Industry in Vietnam: sluggish sector in a growing market. Int J Environ Res Public Health.

[12] World Health Organization. Global tuberculosis report 2019. Geneva World Health Organization, 2019.

[13] Pollack LA, van Santen KL, Weiner LM, Dudeck MA, Edwards JR, Srinivasan A (2016). Antibiotic stewardship programs in U.S. Acute care hospitals: findings from the 2014 national healthcare safety network annual hospital survey. Clin Infect Dis Off Publ Infect Dis Soc Am.

[14] Imwong M, Hien TT, Thuy-Nhien NT, Dondorp AM, White NJ (2017). Spread of a single multidrug resistant malaria parasite lineage (PfPailin) to Vietnam. Lancet Infect Dis.

[15] Anh LV, Lan LT, Huyen HTK (2013). Survey on the vancomycin utilization in Bach Mai hospital. Pharm J (Vietnam).

[16] Song JH, Jung SI, Ko KS (2004). High prevalence of antimicrobial resistance among clinical Streptococcus pneumoniae isolates in Asia (an ANSORP study). Antimicrob Agents Chemother.

[17] Kinh LN, Ha NTB, All E (2011). Antibiotic resistance in hospital infections in intensive care units in some hospitals. Pharm J (Vietnam).

[18] Nhat NK, Huyen HTK (2012). Study on the selection and use of antibiotics in pneumonia treatment in Department of Internal Medicine—Hue Central Hospital from January 2009 to August 2010. Pharm J (Vietnam).

[19] Malani AN, Richards PG, Kapila S, Otto MH, Czerwinski J, Singal B (2013). Clinical and economic outcomes from a community hospital's antimicrobial stewardship program. AJIC Am J Infect Control.

[20] Ministry of Health of Vietnam. National action plan on combating drug resistance in the period from 2013—2020. In: Ministry of Health, editor. Hanoi, Vietnam: Ministry of Health; 2013.

[21] World Health Organization (2007). Everybody’s business: strengthening health systems to improve health outcomes: WHO's framework for actions.

[22] Chuc NT, Larsson M, Falkenberg T, Do NT, Binh NT, Tomson GB (2001). Management of childhood acute respiratory infections at private pharmacies in Vietnam. Ann Pharmacother.

[23] Larsson M, Kronvall G, Chuc N (2000). Antibiotic medication and bacterial resistance to antibiotics: a survey of children in a Vietnamese community. Trop Med Int Health.

[24] Pham TH, Ha NTH, Dien TM, Nguyen TLH (2018). Assessment of primary antibiotics seclection in community pneumonia inpatients in National Pediatric Hospital. Pharm J.

[25] Khanh VH, Hien NT, Hoa VD (2018). Analysis of colistin use in anesthesia and resuscitation center of Viet Duc hospital. Vietnam Pharm J.

[26] Khiem NH, Cuong TM, Van PTT (2017). Analysis of antibiotic use in patients with mechanical ventilation. Pharm J (Vietnam).

[27] Minh NT, Tuyen NT, Thang TN (2017). Survey on the consumption of carbapenem in Bach Mai Hospital in the period of 2012 to 2016. Pharm J (Vietnam).

[28] Tho TTA, Sinh CT (2015). Evaluation of antibiotic use in treatment of pneumonia in children from 2 months to 5 years at Nghe An Obstetrics and Pediatric hospital. Pharm J (Vietnam).

[29] Thuy NTT, Nguyen NT (2017). Survey on knowledge, attitudes and behaviors of using antibiotics by pharmacy customers in urban districts of Ho Chi Minh City. Vietnam Pharm J.

[30] Cuong TQ, Mai HT, Minh NTH (2016). Situational analysis of the risks from drugs affecting human health in the period 2008–2015. Pharm J (Vietnam).

[31] Huy NB, Phung PT, Hoa NM, Hoa VD, Anh NH (2018). Analysis of antibiotic use in patients with hospital pneumonia/ mechanical ventilation pneumonia at intensive care unit, Can Tho General Hospital. Vietnam Pharm J.

[32] Son NT, Tra TT, Thao PTN (2017). Antimicrobial Stewardship Program at a tertiary teaching hospital in Vietnam: a longitudinal observational study. Clin Microbiol Infect Dis.

[33] Wertheim H, Chandna A, Phu V (2013). Providing impetus, tools and guidance to strengthen national capacity for antimicrobial stewardship in Viet Nam. PLoS Med.

[34] Zellweger RM, Carrique-Mas J, Limmathurotsakul D, Day NPJ, Thwaites GE, Baker S (2017). A current perspective on antimicrobial resistance in Southeast Asia. J Antimicrob Chemother.

[35] Thu TA, Rahman M, Coffin S, Harun-Or-Rashid M, Sakamoto J, Hung NV (2012). Antibiotic use in Vietnamese hospitals: a multicenter point-prevalence study. Am J Infect Control.

[36] Chalker J, Chuc N, Falkenberg T, Tomson G (2002). Private pharmacies in Hanoi, Vietnam: a randomized trial of a 2-year multi-component intervention on knowledge and stated practice regarding ARI, STD and antibiotic/steroid requests. Trop Med Int Health.

[37] Diep TS. Antibiotic resistance in the Hospital for tropical diseases. In: GARP workshop, Ho Chi Minh City; 2009.

[38] The National Assembly. Law on pharmacy. In: The National Assembly of Vietnam (ed) 105/2016/QH13. The National Assembly of Vietnam, Hanoi, Vietnam; 2016.

[39] The National Assembly. Law on medical examination and treatment. In: The National Assembly of Vietnam (ed) 40/2009/QH12. Hanoi, Vietnam: The National Assembly of Vietnam; 2009.

[40] Vietnam Government. Decree No 54/2017/ND-CP to provide guidelines for implementation of the Law on Pharmacy. In: The Government (ed). Vietnamese Government, Hanoi, Vietnam; 2017.

[41] Vietnam Government. Decree 176/2013/ND-CP on penalties for administrative violations against medical laws. In: The Government (ed) Vietnamese Government, Hanoi, Vietnam; 2013.

[42] Vietnam Government. Decree 155/2018/ND-CP to amend and supplement a number of regulations related to business investment conditions under the state management scope of the Ministry of Health. In: The Government (ed) Vietnamese Government, Hanoi, Vietnam; 2018.

[43] Ministry of Health of Vietnam. Circular 02/2018/TT-BYT providing guidelines for Good Pharmacy Practices. In: Vietnam DAo (ed). Ministry of Health, Vietnam; 2018.

[44] Ministry of Health of Vietnam. Circular No 46/TT-BYT on the principles and standards of a Good Pharmacy Practice; 2011.

[45] Ministry of Health of Vietnam. Circular No 21/2013/TT-BYT on the organization and activities of Drug and Treatment Council in hospital; 2013.

[46] Ministry of Health of Vietnam. Circular No 33/2016/TT-BYT on the organization and activities of microbiology testing in hospital; 2016.

[47] Ministry of Health of Vietnam. Circular 31/2012/TT-BYT on Clinical pharmacy activities in hospitals; 2012.

[48] Ministry of Health of Vietnam. Circular No 07/VBHN-BYT date April 19 2018 on drug use guideline for health facilities with patient beds; 2018.

[49] Ministry of Health of Vietnam. Circular No 16/2018/TT-BYT on infection control in the examination and treatment facilities; 2018.

[50] Ministry of Health of Vietnam. Circular 38/2010/TT-BYT guiding on monitoring activities for compliance with state regulations on pharmacy and cosmetic; 2010.

[51] Ministry of Health of Vietnam. Circular 22/2013/TT-BYT on continuous training for health professional. In: Training AoSTa (ed) 2013.

[52] Ministry of Health of Vietnam. Circular 07/2017/TT-BYT on promulgation of list of OTC drugs. In: Vietnam DAo (edi) 2017.

[53] Ministry of Health of Vietnam. Circular 52/2017/TT-BYT: regulation for prescription of drugs and biologicals in outpatient treatment. In: Administration MS (ed) Vietnam; 2017.

[54] Ministry of Health of Vietnam. Decision 11/2007/QD-BYT on the principles and standards of Good Pharmacy Practice; 2007.

[55] Ministry of Health of Vietnam. Decision 6211/QD-BYT dated October 17 2016 on setting up and defining the functions and tasks of the drug-related resistance surveillance network in medical examination and treatment facilities; 2016.

[56] Ministry of Health of Vietnam. Decision No 4324/QD-BYT September 26 2017 to establish the Steering Committee to implement Project Enhancing the prescription control and prescription medicine in the period of 2017–2020; 2017.

[57] Ministry of Health of Vietnam. Decision No. 2888/QD-BYT dated 05 August 2014 on establishment of sub-committees on AMR supervision; 2014.

[58] Ministry of Health of Vietnam. Decision No 3861/QD-BYT dated September 30 103 defining the functions, tasks, powers and organizational structure of Drug Administration of Vietnam; 2013.

[59] Ministry of Health of Vietnam. Decision No 3391/QD-BYT dated August 14 2015 on the establishment of National AMR monitoring Unit; 2015.

[60] Ministry of Health of Vietnam. Decision 5888/QD-BYT dated October 10 2016 on establishment of National Steering Committee on AMR in the period of 2016 to 2020; 2016.

[61] Ministry of Health of Vietnam. Decision No 4518/QD-BYT dated July 16 2018 defining the functions, tasks, powers and organizational structure of Medical Service Administration; 2018.

[62] Ministry of Health of Vietnam. Decision 2418/QD-BYT dated April 10 2018 defining the functions, tasks, powers and organizational structure of Department of Health Insurance of MoH; 2018.

[63] Vietnam Prime Minister. Directive No. 23/CT-TTg on strengthening the management and linkages of drug supply facilities. In: Government V (ed) Vietnamese Government, Hanoi, Vietnam; 2019.

[64] Ministry of Health of Vietnam. Guidance on management of antibiotic use in hospitals issued under Decision 772/QD-BYT dated March 4 2016; 2016.

[65] Ministry of Health of Vietnam & Health Partner. Joint annual health review 2008 (Chapter I: Update on the health sector); 2008.

[66] Ministry of Health of Vietnam & Development Partner. Joint annual health review; 2016.

[67] Ministry of Health of Vietnam & Development Partner. Joint annual health review; 2015.

[68] Drug Administration of Vietnam. Report on applying information technology in connecting the drug supply facilities; 2018.

[69] MOH Medical Service Administration. Implementing national action plan on AMR prevention, 2017.

[70] MOH Administration of Science Technology and Training. Health professional education to meet the requirement of difficulty areas; 2016.

[71] Hoang Hieu Tri. Pharmaceutical sector report; 2014.

[72] HCMC Department of Health. Implementing the action plan on AMR in Ho Chi Minh City Conference on Preliminary Results of Implementing National Action Plan to Combat AMR in Vietnam. Ho Chi Minh City; 2017.

[73] Cho Ray hospital. Antibiotic use surveillance in Cho Ray hospital. In: Conference on preliminary results of implementing national action plan to combat AMR in Vietnam. Ho Chi Minh City; 2017.

[74] National Hospital for Tropical Diseases. Implementing results of antimicrobial resistance supervision program. In: Conference on preliminary results of implementing national action plan to combat AMR in Vietnam. Ho Chi Minh City; 2017.

[75] Saint Paul Hospital. Antimicrobial resistance prevention in Saint Paul hospital. In: Conference on preliminary results of implementing national action plan to combat AMR in Vietnam. Ho Chi Minh City; 2017.

[76] Le Nguyen Hai Anh. Study on the expenditure of health insurance medicine of some health facilities in provinces during 2014–2015, 2017.

[77] MOH Medical Service Administration. Report on hospital checking 2010; 2010.

[78] MOH M, MARD, MRE and development partners. Multi-sector commitment on AMR prevention and control; 2015.

[79] World Health Organization. WHONET Software; 2020. https://www.who.int/medicines/areas/rational_use/AMR_WHONET_SOFTWARE/en/. Accessed 23 March 2020.

[80] Zawahir S, Le HTT, Nguyen T-A (2022). Inappropriate supply of antibiotics for common viral infections by community pharmacies in Vietnam: a standardised patient survey. Lancet Reg Health Western Pacific.

[81] Zawahir S, Le H, Nguyen TA (2021). Standardised patient study to assess tuberculosis case detection within the private pharmacy sector in Vietnam. BMJ Glob Health.

[82] Beardsley J, Chambers JM, Lam TT (2023). Mapping access to drug outlets in Vietnam: distribution of drug outlets and the sociodemographic characteristics of the communities they serve. Lancet Reg Health Western Pac.

[83] Do NTT, Vu HTL, Nguyen CTK (2021). Community-based antibiotic access and use in six low-income and middle-income countries: a mixed-method approach. Lancet Glob Health.

[84] Nguyen NV, Do NTT, Nguyen CTK (2020). Community-level consumption of antibiotics according to the AWaRe (Access, Watch, Reserve) classification in rural Vietnam. JAC-Antimicrob Resist.

[85] Vu TVD, Do TTN, Rydell U (2019). Antimicrobial susceptibility testing and antibiotic consumption results from 16 hospitals in Viet Nam: The VINARES project 2012–2013. J Globl Antimicrob Resist.

[86] The World Bank. Current health expenditure per capita; 2019. https://data.worldbank.org/indicator/SH.XPD.CHEX.PP.CD?locations=VN. Accessed 18 Mar 2022.

[87] Health Policy and Strategy Institute, Drug Administration of Vietnam, World Health Organization. National Medicines Policy Assessment; 2010.

[88] Nga Do TT, Chuc NT, Hoa NP (2014). Antibiotic sales in rural and urban pharmacies in northern Vietnam: an observational study. BMC Pharmacol Toxicol.

[89] van Doorn R, Miliya T, Douangnouvong A, et al. A Clinically Oriented antimicrobial Resistance surveillance Network (ACORN): pilot implementation in three countries in Southeast Asia, 2019–2020. Wellcome Open Res; 2022. 10.12688/wellcomeopenres.18317.1 PMC10579863 37854668

[90] World Health Organization (2017). Global antimicrobial resistance surveillance system (GLASS) report—early implementation 2016–2027.

[91] Ventola CL (2015). The antibiotic resistance crisis: part 2: management strategies and new agents. P&T Peer-reviewed J Formul Manag.

[92] Cantey JB, Vora N, Sunkara M (2017). Prevalence, characteristics, and perception of nursery antibiotic stewardship coverage in the United States. J Pediatr Infect Dis Soc.

[93] Zhou J, Ma X (2019). A survey on antimicrobial stewardship in 116 tertiary hospitals in China. Clin Microbiol Infect Off Publ Eur Soc Clin Microbiol Infect Dis.

[94] Ahiabu M-A, Magnussen P, Bygbjerg IC, Tersbøl BP (2018). Treatment practices of households and antibiotic dispensing in medicine outlets in developing countries: the case of Ghana. Res Soc Adm Pharm.

[95] Doern GV, Pfaller MA, Kugler K, Freeman J, Jones RN (1998). Prevalence of antimicrobial resistance among respiratory tract isolates of Streptococcus pneumoniae in North America: 1997 results from the SENTRY antimicrobial surveillance program. Clin Infect Dis Off Publ Infect Dis Soc Am.

[96] Haines A, Kuruvilla S, Borchert M (2004). Bridging the implementation gap between knowledge and action for health. Bull World Health Organ.

[97] World Health Organization (2018). Global antimicrobial resistance surveillance system (GLASS) report: early implementation.

[98] Doron S, Nadkarni L, Lyn Price L (2013). A nationwide survey of antimicrobial stewardship practices. Clin Ther.

[99] Broom A, Plage S, Broom J, Kirby E, Adams J (2016). A qualitative study of hospital pharmacists and antibiotic governance: negotiating interprofessional responsibilities, expertise and resource constraints. BMC Health Serv Res.

[100] Thakolkaran N, Shetty AV, D'Souza NDR, Shetty AK (2017). Antibiotic prescribing knowledge, attitudes, and practice among physicians in teaching hospitals in South India. J Fam Med Prim Care.

[101] Blanchette L, Gauthier T, Heil E (2018). The essential role of pharmacists in antibiotic stewardship in outpatient care: an official position statement of the Society of Infectious Diseases Pharmacists. J Am Pharm Assoc JAPhA.

[102] Heil EL, Kuti JL, Bearden DT, Gallagher JC (2016). The essential role of pharmacists in antimicrobial stewardship. Infect Control Hosp Epidemiol.

[103] Essack S, Bell J, Shephard A (2018). Community pharmacists-Leaders for antibiotic stewardship in respiratory tract infection. J Clin Pharm Ther.

[104] Belkina T, Al Warafi A, Hussein Eltom E, Tadjieva N, Kubena A, Vlcek J (2014). Antibiotic use and knowledge in the community of Yemen, Saudi Arabia, and Uzbekistan. J Infect Dev Ctries.

[105] Gebretekle GB, Haile Mariam D, Abebe W (2018). Opportunities and barriers to implementing antibiotic stewardship in low and middle-income countries: lessons from a mixed-methods study in a tertiary care hospital in Ethiopia. PLoS ONE.

[106] Jamhour A, El-Kheir A, Salameh P, Hanna PA, Mansour H (2017). Antibiotic knowledge and self-medication practices in a developing country: a cross-sectional study. AJIC Am J Infect Control.

[107] Molstad S, Lofmark S, Carlin K (2017). Lessons learnt during 20 years of the Swedish strategic programme against antibiotic resistance. Bull World Health Organ.

[108] Pulcini C, Binda F, Lamkang AS (2019). Developing core elements and checklist items for global hospital antimicrobial stewardship programmes: a consensus approach. Clin Microbiol Infect.

[109] Joshi MP, Chintu C, Mpundu M (2018). Multidisciplinary and multisectoral coalitions as catalysts for action against antimicrobial resistance: implementation experiences at national and regional levels. Glob Public Health.

