# Supplementary material for: Health system barriers to the implementation of the national action plan to combat antimicrobial resistance in Vietnam: a scoping review
Source: Antimicrob Resist Infect Control. 2024 Jan 25;13:12. doi: 10.1186/s13756-024-01364-x (PMC10809436; doi:10.1186/s13756-024-01364-x)
Supplement: Supplementary file 1 — Additional file 1. Search strategies. [file 13756_2024_1364_MOESM1_ESM.docx]

**Table S1. Search strategies**

| Search | Query | Hits |
| --- | --- | --- |
| #1 | "Anti-Bacterial Agents"[Mesh] OR "Anti-Bacterial Agents" [Pharmacological Action] OR (antibacterial [All Fields] AND agents [All Fields]) OR anti-bacterial agents [All Fields] OR antibiotics [All Fields] OR antimicrobial [All Fields] AND ("Drug Resistance, Microbial"[Mesh] OR "Antimicrobial Stewardship"[Mesh] OR "Microbial Sensitivity Tests"[Mesh]) OR (“Antimicrobial” [All Fields] AND “stewardship” [All Fields]) OR antibiotic resistance | 109,827 |
| #2 | "Delivery of Health Care"[Mesh] OR "Health Information Systems"[Mesh] OR "Delivery of Health Care, Integrated"[Mesh] OR "Community Health Planning"[Mesh] OR "Health Information Interoperability"[Mesh] OR "Health Resources"[Mesh] OR "Workforce"[Mesh] | 61,086 |
| #3 | "Developed Countries"[Mesh] OR "Developing Countries"[Mesh] OR low and middle income countr* OR resources limited countr* OR resources constrained countr* OR non-industrialized countr* | 273,481 |
| #4 | #1 AND #2 AND #3 | 158 |

Retained after title and abstract screening (irrelevant, duplicates, not in English and can’t access full-texts removed): 48

Retained after full text screening: 22
